# Biodiversity of the antimicrobial potential of *Weissella paramesenteroides* strains isolated from dairy cattle

**DOI:** 10.3389/fmicb.2026.1750102

**Published:** 2026-02-20

**Authors:** Sebastian W. Fischer, Anna Euler, Leonie Bertels, Nadine Mariani Corea, Fritz Titgemeyer

**Affiliations:** 1 Institute for Hygiene and Public Health, University Clinics Bonn, Bonn, Germany; 2 Department of Food - Nutrition - Facilities, FH Muenster, Münster, Germany

**Keywords:** antibacterial, biodiversity, food safety, genome fluidity, genome plasticity, lactic acid bacteria, *Weissella*

## Abstract

*Weissella* species are lactic acid bacteria with a high potential for the fermentation of food items. They ferment fruits, vegetables, fish, and meat to deliver aromatic ingredients, produce antibacterial and antifungal compounds, and exert probiotic properties. Despite these attractive attributions, they are still poorly studied. We have isolated 40 strains of *Weissella paramesenteroides* from one biotope, the udder of milk cattle, to examine genomic plasticity, antibacterial efficacy, and organic acid and hydrogen peroxide formation. Each isolate was identified by DNA sequence comparisons of the 16S rRNA-encoding gene. Patterns of genomic DNA fragments from random amplification of polymorphic DNA showed that 38 isolates differed by more than 5%, thus representing subspecies. Nine isolates were selected for further characterization. They were able to inhibit all eight food-borne pathogens and surrogates used. *Salmonella enterica* and *Pseudomonas aeruginosa* were strongly inhibited, while inhibition of *Listeria monocytogenes* was weaker but still significant. However, the inhibition profiles varied considerably. Acid production was assessed for each isolate in the presence of indicator strains exhibiting different acidification patterns as well. The presence of *S. enterica* was associated with a drop in pH, whereas no notable acidification occurred when isolates were challenged with *Klebsiella pneumoniae* and *Bacillus subtilis*. To confirm the observed differences, we analyzed the pan-genomes of 10 genomes retrieved from the NCBI database. Each genome contains several 100 accessory genes and up to 6.5% unique genes, indicating a high genome fluidity and additional strain-specific metabolic capacity. We suggest that the antibacterial efficacy of *W. paramesenteroides* is based on a multi-barrier system, whereby strains have developed different genetic qualities for the expression of an individual set of barriers.

## 1 Introduction

*Weissella* are Gram-positive, non-motile, facultative anaerobic microorganisms that grow heterofermentatively (Teixeira et al., 2021b; Fusco et al., 2023). They are closely related to *Periweissellae*, *Furfurilactobacilli*, and *Leuconostocha* (Qiao et al., 2023). The 26 currently described *Weissella* species belong to the order *Lactobacillales*, comprising 36 genera and over 400 species (Zheng et al., 2020; Bello and Gupta, 2025; Göker et al., 2025). Phylogenetic analyses have classified them into six species groups, each defined by a signature species: *Weissella beninensis*, *Weissella kandleri*, *Weissella oryzae*, *Weissella halotolerans*, *Weissella confusa*, and *Weissella paramesenteroides* (Fanelli et al., 2022). According to the PubMed database, members of the *W. confusa* and *W. paramesenteroides* groups, including *W. cibaria*, *W. confusa*, and *W. paramesenteroides*, have been predominantly investigated, with 440, 343, and 128 mentions in titles and abstracts, respectively. However, they are less well studied, compared to some well-characterized *Lactobacillus* species or closely related species such as *Leuconostoc mesenteroides*, which have several 1,000 entries in PubMed (Apostolakos et al., 2022).

*Weissella* species are ubiquitous. They are found among others in soil, tomatoes, bee pollen, papaya, the rhizosphere of olive trees, Chardonnay grapes, red pepper, desert plants, milk, dairy, kimchi, sourdough, fermented fish, fermented sausages, Mexican podzol, and especially in fermented beverages and fruits, and even in heroin (Fusco et al., 2015; Onur and Önlü, 2023). In humans, the oral cavity, breast milk, the gastrointestinal tract, the vagina, and feces are places where *Weissella* species occur (Walter et al., 2001; Martín et al., 2007a, 2007b; Albesharat et al., 2011; Kang et al., 2011; Teixeira et al., 2021a).

As gut-dwelling bacteria, they contribute to human health. This encompasses the degradation of dietary fibers, secretion of folic acid, anti-inflammatory effects, and immune system stimulation (Xiong et al., 2025). It has further been shown that *W. confusa* and *W. cibaria* produce exopolysaccharides and thus deliver prebiotic nutrients for beneficial gut-dwelling bacteria. *Weissella* species can tolerate low pH values, bile salts, and can adhere to the epithelial cells of the gut mucosa, three advantageous features to reside in the gastrointestinal tract (Pabari et al., 2020; Teixeira et al., 2021a; Onur and Önlü, 2023). Another characteristic is the inhibition of pathogens (Agirman et al., 2021). The mechanisms comprise the secretion of organic acids, nutrient and epithelial adhesion-site competition, hydrogen peroxide (H_2_O_2_) formation under oxygen stress, disruption of quorum-sensing communication of target bacteria, secretion of bacteriocins and small peptides, and so-called bacteriocin-like inhibitory substances (BLIS) (Papagianni and Papamichael, 2011; Silva et al., 2018; Fischer and Titgemeyer, 2023; Singh et al., 2024; Thuy et al., 2024; Rotta et al., 2025). *Weissella* species can inhibit foodborne pathogens like *Bacillus cereus*, *Escherichia coli*, *Klebsiella pneumoniae, Listeria monocytogenes*, *Pseudomonas aeruginosa*, *Salmonella enterica*, and *Staphylococcus aureus,* as well as various fungi, particularly by ion scavenging (Papagianni and Papamichael, 2011; Teixeira et al., 2021a; Wan et al., 2023; Thuy et al., 2024).

The abovementioned features, in combination with good growth capacities under standard laboratory conditions, indicate a favorable use of *Weissella* species in food items (Fessard and Remize, 2017). The frequent isolation from diverse biotopes points toward a rich, exploitable gene pool (Fusco et al., 2015).

Here, we report on an analysis of *W. paramesenteroides* strains isolated from foremilk and teat canal biofilms from dairy cows. We describe the genetic diversity and show differences in antibacterial efficacy and acid production against a range of foodborne pathogens and surrogates. To further explain the observed differences, we analyzed the genomic fluidity by examination of available completed *W. paramesenteroides* genomes retrieved from the NCBI genome database.

## 2 Materials and methods

### 2.1 Strain isolation and indicator strains

Samples were obtained from 18 farms situated in Münsterland, Germany. They were collected as follows. The udder of a cow was disinfected with an alcohol-soaked cloth before a biofilm was collected by introducing a swab for about 2 cm into the teat canal. Foremilk samples of 5–10 mL were withdrawn by milking. Samples were diluted in 0.9% sodium chloride solution and propagated as described previously (Mariani Corea et al., 2025). The isolated colonies were subjected to polymerase chain reactions (PCR) using primer 27f and 535R of the 16S rRNA gene (Fischer et al., 2025). The PCR products were subsequently sequenced by Microsynth Seqlab (Germany). Identification of species was achieved by using the Basic Local Alignment Tool (BLAST) provided by the National Center for Biotechnology Information (NCBI, United States) (Altschul et al., 1990). The isolates of *W. paramesenteroides* strains used in this study are listed in Table 1.

**Table 1:** List of *W. paramesenteroides* isolates.

No.	Strain	Farm	Animal husbandry, sample
1	05.3 92.2 VL-3	1	Conventional, biofilm
2	05.3162.2 HL-2^1^	1	Conventional, biofilm
3	05.3162.2 HL-3^1^	1	Conventional, biofilm
4	13.7 1A-4	13	Conventional grazing, biofilm
5	13.7 6A-9	13	Conventional grazing, biofilm
6	13.7 10A-2	13	Conventional grazing, biofilm
7	13.7 11A-5	13	Conventional grazing, biofilm
8	13.7 13A-1	13	Conventional grazing, biofilm
9	13.7 14A-1	13	Conventional grazing, biofilm
10	13.7 17–2	13	Conventional grazing, foremilk
11	13.7 19A-10	13	Conventional grazing, biofilm
12	13.7 21A-7	13	Conventional grazing, biofilm
13	13.7 22A-1^2^	13	Conventional grazing, biofilm
14	13.7 22A-2^2^	13	Conventional grazing, biofilm
15	13.7 22A-5^2^	13	Conventional grazing, biofilm
16	13.7 22A-7^2^	13	Conventional grazing, biofilm
17	18.5 1A-3	7	Conventional, biofilm
18	18.5 11–4	7	Conventional, foremilk
19	18.5 12A-10	7	Conventional, biofilm
20	18.5 19–3	7	Conventional, foremilk
21	20.4 7A-5^3^	3	Organic, biofilm
22	20.4 7A-6^3^	3	Organic, biofilm
23	22.4 S2-4	4	Collection tank
24	22.4 6A-2	4	Conventional, biofilm
25	22.4 7–3	4	Conventional, foremilk
26	22.4 7A-9	4	Conventional, biofilm
27	22.4 12A-3	4	Conventional, biofilm
28	26.3 5A-2	2	Conventional, biofilm
29	26.3 6A-5	2	Conventional, biofilm
30	27.4 5–4	4	Conventional, foremilk
31	27.4 6–1	4	Conventional, foremilk
32	27.4 8–1	4	Conventional, foremilk
33	27.8 11A-2^4^	18	Conventional, biofilm
34	27.8 11A-5^4^	18	Conventional, biofilm
35	27.8 11A-6^4^	18	Conventional, biofilm
36	27.8 13A-4	18	Conventional, biofilm
37	31.5 4A-4	10	Conventional, biofilm
38	31.5 18–1^5^	10	Conventional, foremilk
39	31.5 18–6^5^	10	Conventional, foremilk
40	31.5 19–6	10	Conventional, foremilk

^1, 2, 3, 4, 5^The isolates were derived from the same quarter of the udder.

Eight bacterial species were employed as indicators and surrogates, representing foodborne pathogens and food spoilage organisms (Table 2). The strains were cultivated in Luria-Bertani (LB) Broth (Carl Roth, Germany) at 37 °C for 24 h. Brain Heart Infusion (BHI) Broth (BD, USA) was utilized for the cultivation of *L. monocytogenes*.

**Table 2:** List of indicator strains.

Species	Origin	Comment
*Bacillus subtilis*	Laboratory strain collection	Surrogate food spoilage
*Citrobacter koseri*	Laboratory strain collection	Fish pathogen
*Klebsiella pneumoniae*	Laboratory strain collection	Opportunistic pathogen
*Listeria monocytogenes*	Dsm 20,600	Foodborne pathogen
*Pseudomonas aeruginosa*	Atcc 15442	Food spoilage and pathogen
Salmonella enterica*	Lt-2	Foodborne pathogen
*Staphylococcus haemolyticus*	Laboratory strain collection	Surrogate food pathogen
*Staphylococcus warneri*	Laboratory strain collection	Surrogate food pathogen

*re-classified from originally *S. typhimurium* to *S. enterica* ssp. enterica ser. Typhimurium (Ames et al., 1975; Judicial Commission of the International Committee on Systematics of Prokaryotes, 2005).

### 2.2 Random-amplified-polymorphic-DNA analysis (RAPD)

RAPD–PCR fingerprinting was carried out by applying the M13 minisatellite core primer (5′-GAGGGTGGCGGTTCT-3′) (Rossetti and Giraffa, 2005). PCRs were conducted as previously described (Fischer et al., 2025). The amplification products were separated by 2% agarose gel electrophoresis (AppliChem, Germany).

### 2.3 Hydrogen peroxide production

Hydrogen peroxide production was conducted as described (Tomás et al., 2004). Colonies were streaked out from an overnight culture onto an mMRS agar plate supplemented with 1 mM 3,3′,5,5′-Tetramethylbenzidin (TMB) and 2 U/mL of type II horseradish peroxidase (Carl Roth, Germany). Agar plates were incubated anaerobically for 48 h at 30 °C before the lid of the Petri dish was removed for 10 min of aeriation. The formation of H_2_O_2_ became visible when the yellowish colonies turned black.

### 2.4 Lawn-on-spot assays and acidification

Antimicrobial activities against foodborne pathogens and food spoilage organisms were evaluated with lawn-on-spot assays as described previously (Mariani Corea et al., 2025). *W. paramesenteroides* isolates were inoculated from a liquid overnight culture by positioning 2 μL into the center of an MRS agar plate supplemented with 0.5 g/L cysteine (Carl Roth, Germany) (mMRS) and incubated under anaerobic conditions at 30 °C for 48 h. A cell suspension of 0.2 mL containing 2.0×10^7^ CFU/mL from a fresh overnight culture of the respective indicator strain was mixed with 10 mL of LB-soft agar (0.8%, Bacto Agar, BD, United States) or BHI-soft agar (0.8%) in the case of *L. monocytogenes*. The suspension was poured onto an mMRS plate. Assay plates were aerobically incubated in co-cultivation for 20–30 h at 37 °C. The clear halo (zone of inhibition), from which the colony diameter was subtracted, was measured in millimeters. Acid secretion of the *W. paramesenteroides* isolates was monitored with color-fixed indicator sticks (pH Strips; Carl Roth). In this way, it was possible to document changes in pH by comparing the pH in the inhibition zone with the pH of the indicator strain grown at the edge of the same agar plate. As a control, the pH was measured for all isolates and indicator strains solely cultivated on mMRS-soft agar plates.

### 2.5 Data analyses and statistics

Data analyses comprised subspecies identification, antimicrobial activity *in vivo*, and the relationship between antimicrobial compounds and acid secretion. Therefore, the statistical and computing package R (v4.2.1) and Rstudio (v2023.03.0 + 386) with packages tidyverse (v2.0.0), readxl (v1.4.2), ggplot2 (v3.4.2), vegan (2.6–4), and scico (v1.5.0) were used (Crameri, 2018; Wickham et al., 2019; R Core Team, 2021; Oksanen et al., 2022; RStudio Team, 2022; Wickham and Bryan, 2022). Subspecies identification was based on the analysis of the data derived from RAPD-PCR DNA band patterns. The unweighted pair group method with arithmetic mean (UPGMA) was applied by using the hclust algorithm together with the Dice dissimilarity coefficient matrix from the R base package (Hilton et al., 1997; Ghazi et al., 2016). Phylogenetic trees were generated using a threshold of ≤5% dissimilarity to distinguish clonal isolates (Ghazi et al., 2016; Szaluś-Jordanow et al., 2018; Ruiz et al., 2014; Nielsen et al., 2014; Birch et al., 1996). Heatmaps showing antibacterial activities and acidification were generated on the basis of the average values from triplicates. Spearman correlation and scatterplot visualization performed on the raw replicate values assessed the contributions of antimicrobial compounds and acid secretion at a 95% confidence level. Phylogenetic trees, heatmaps, and scatterplots were visualized using ggplot2.

### 2.6 Genome analyses

All available complete genomes of *W. paramesenteroides* together with the respective plasmids, were retrieved from the NCBI genome database: GCF_050613345.1, GCF_051903945.1, GCF_002386265.1, GCF_028994215.1, GCF_045005335.1, GCF_045006675.1, GCF_036327715.1, GCF_015689215.1, GCF_030168775.1, and GCA_052281005.1. Venn diagrams showing pan-genomes were computed with EDGAR 3.0 on the basis of the gene annotation list created with Bakta (Dieckmann et al., 2021; Schwengers et al., 2021). Singleton gene calculation was done with EDGAR 3.0 by running each of the 10 genomes against the remaining nine (Dieckmann et al., 2021). Genome screening of bacteriocin-encoding genes was performed using webserver BAGEL4 and BLASTP of the National Center for Biotechnology Information (Altschul et al., 1990; van Heel et al., 2018).

## 3 Results

### 3.1 Isolation of *Weissella paramesenteroides* strains and genetic biodiversity

A screening for lactic acid bacteria (LAB) from teat canal biofilms and foremilk samples from lactating cows across 18 dairy farms in Münsterland, Germany, yielded several 100 cultivable isolates. Species were assigned by BLASTN database alignments of the sequenced 16S rRNA gene fragments. Forty unequivocally identified isolates of *W. paramesenteroides*, which represented a prominent, well-cultivable group, were then selected for this study.

**Figure 1:**
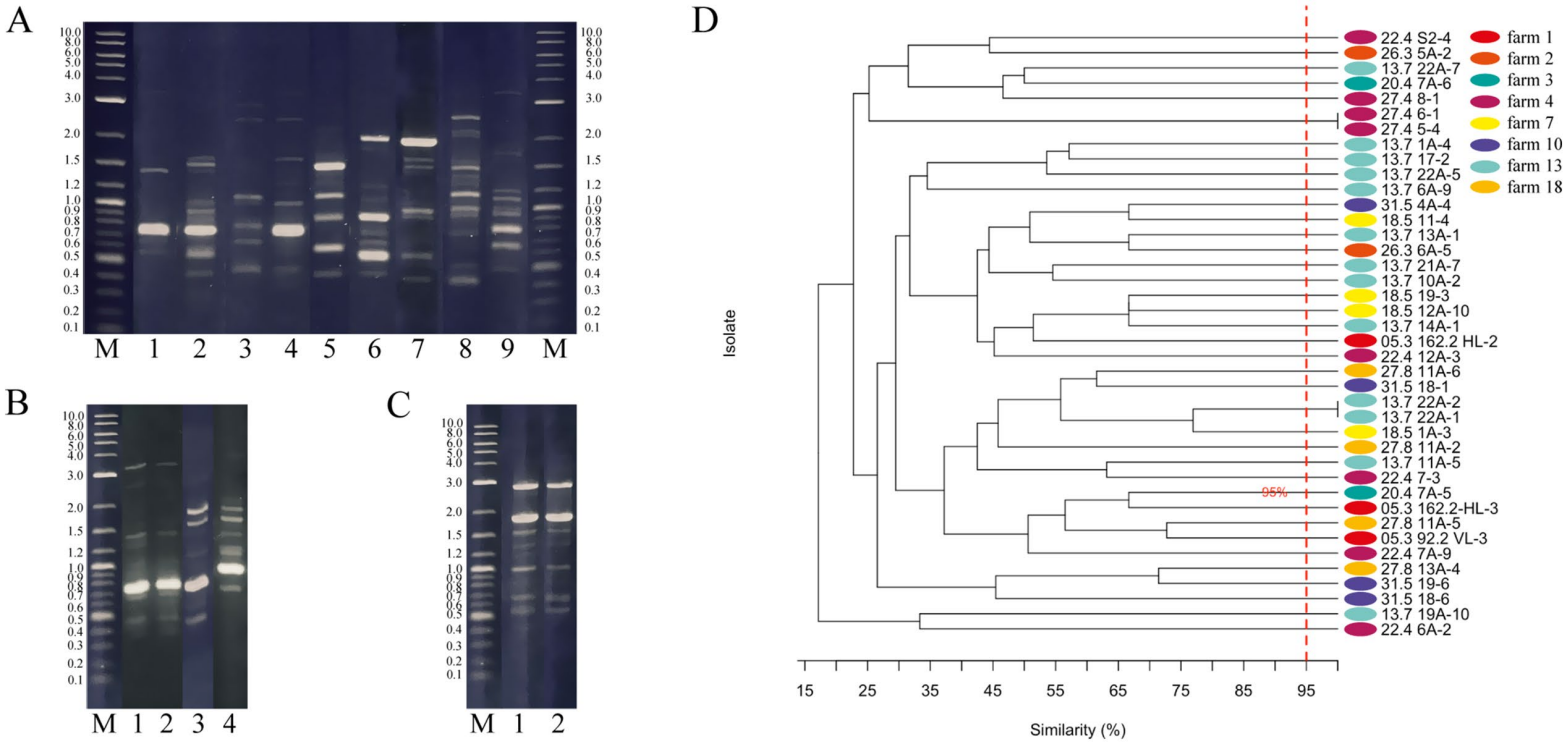
RAPD fingerprints and UPGMA dendrogram. **(A–C)** Shown are 2% agarose gels with **(A)** the isolates that were used for further analyses: M, DNA Marker; 1, 05.3 162-HL-2; 2, 05.3 162-HL-3; 3, 13.7 17 -2; 4, 13.7 19A-10; 5, 20.4 7A-5; 6, 20.4 7A-6; 7, 22.4 7A-9; 8, 22.4 6A-2; 9, 22.4 S2-4. **(B)** Four *W. paramesenteroides* isolates were isolated from the same teat, of which 1 and 2 are identical: M, DNA Marker; 1, 13.7 22A-1; 2, 13.7 22 A-2; 3, 13.7 22A-5; and 13.7 22A-7. **(C)** Shown are the two identical isolates from the same farm isolated from different cows: M, DNA Marker; 1, 27.4 5–4; 2, 27.4 6–1. **(D)** UPGMA dendrogram depicting the relative relatedness (%) of the Weissella isolates. The red line shows the 95% threshold that was suggested to define subspecies (Nielsen et al., 2014). Data were obtained from duplicates.

To determine whether *W. paramesenteroides* isolates differed genetically or represented clones, each strain was subjected to RAPD experiments. As shown in Figure 1A, differences in the gene fragment patterns were obvious between most isolates. The derived UPGMA dendrogram resolved multiple well-defined clusters alongside long-branch outliers (Figure 1D). Two pairs of isolates (27.4 6–1, 27.4 5–4, 13.7 22A-1, and 13.7 22A-2) co-clustered at 100% similarity, indicating clonal or near-clonal RAPD fingerprints (Figures 1B–D). The next highest grouping occurred near 75% similarity. Others resolved at the dendrogram periphery with markedly low relatedness to the remainder, reflecting more pronounced intraspecific divergence. Hence, 38 isolates represented subspecies as has been defined by a threshold of <95% (Figure 1D). Nine isolates were selected for further analyses.

### 3.2 Antimicrobial activity spectra

**Figure 2:**
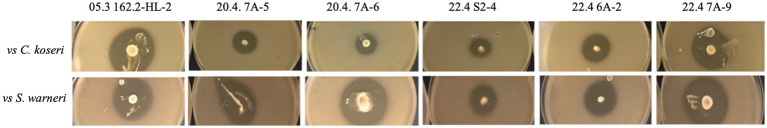
Zones of inhibition of six *W. paramesenteroides* isolates versus *C. koseri* and *S. warneri*. It shows the different sizes of the inhibition zones of the isolates against *C. koseri* and *S. warneri* and size differences by each isolate co-cultured with the respective indicator strain.

**Figure 3:**
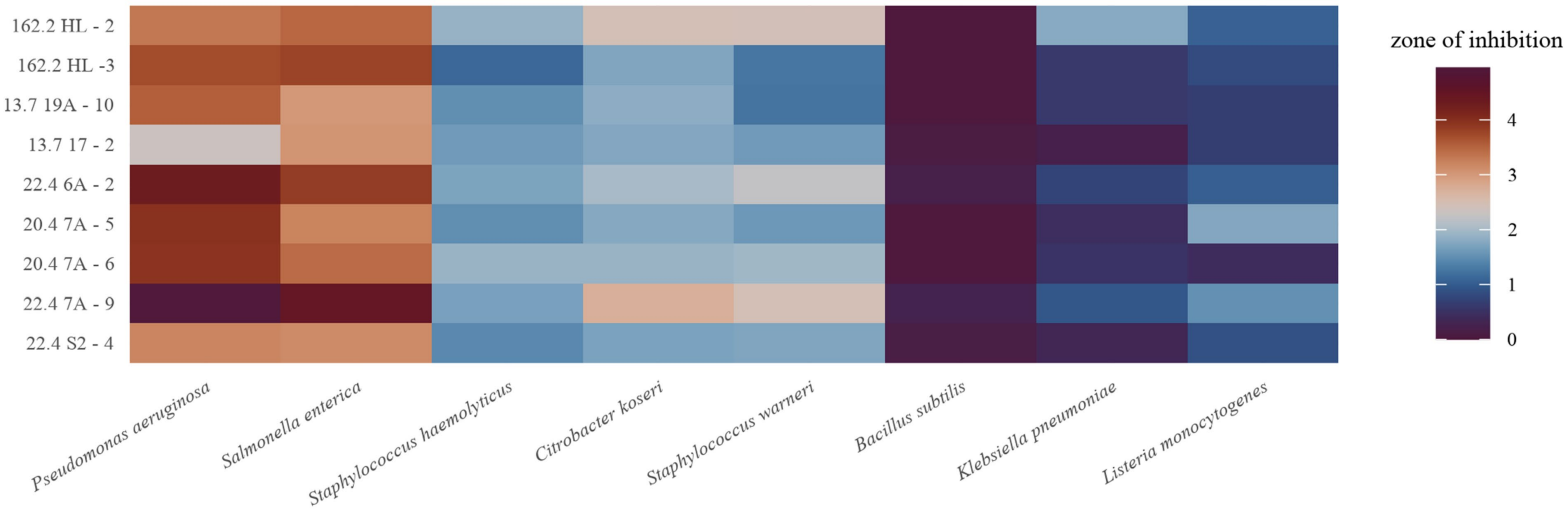
Heatmap of antibacterial activities. The color scale shows the zones of inhibition in cm. Experiments were carried out in triplicate.

Antibacterial activities were assessed by lawn-on-spot assays challenging each *Weissella* isolate against a selection of Gram-negative and Gram-positive indicator strains representing foodborne pathogens and foodborne surrogates (Table 2). The diameters of the zones of inhibition varied between none and 4.9 cm. The differences in halo sizes are illustrated for six isolates against the Gram-negative *C. koseri* and the Gram-positive *S. warneri* (Figure 2). It can be seen that the isolates inhibited the indicators differently, and that the indicators also showed differences in halo size compared to the same isolate, as can be deduced for isolate 20.4 7A-6. A heatmap was calculated showing that all isolates were able to inhibit the indicators, with the exception of *B. subtilis,* which was weak but significantly inhibited by four isolates (Figure 3). The antimicrobial profiles were quite heterogeneous across all combinations. In general, the isolates performed best against the Gram-negatives *P. aeruginosa* and *S. enterica*, while *K. pneumoniae* was less sensitive. Several isolates, particularly 22.4 7A-9 and 05.3162.2 HL-2, displayed robust, broad-spectrum inhibition (Figure 3 and Table 3). In contrast, isolate 13.7 17–2 was the weakest isolate overall. In summary, the data presented in the heatmap reveal a broad but subspecies-specific antibacterial potential within the *W. paramesenteroides* collection of isolates. They will allow the systematic selection of the actual best choice against one, two, or any combination of indicators.

### 3.3 Acidification and hydrogen peroxide production

**Figure 4:**
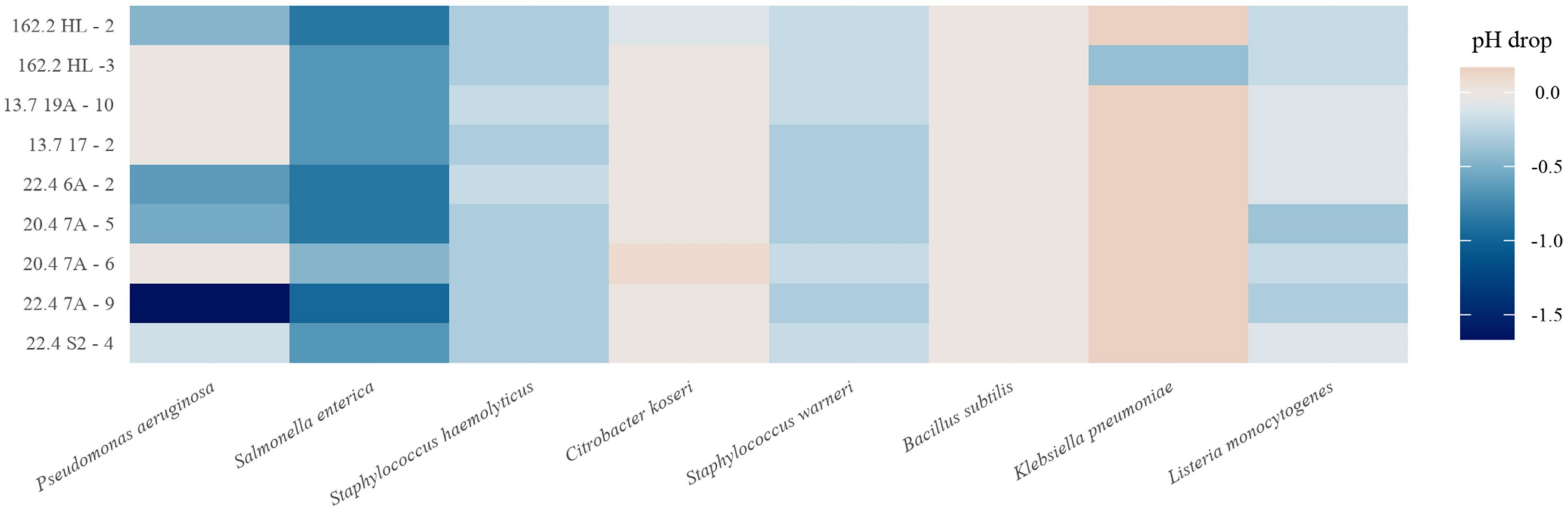
Heatmap of pH drop of each *W. paramesenteroides* isolate in co-culture with each indicator. Data represent the average of triplicates.

We also raised the question of whether the production of organic acids or H_2_O_2_ correlates with antibacterial activity. Acidification by the isolates was monitored in all lawn-on-spot assays by measuring the pH drop between the inhibition zone and the edge of the agar plate. The heatmap shown in Figure 4 displays to which extent the isolates could acidify the medium. Interestingly, this was variable and influenced by the presence of the respective indicator strain. While 22.4 7A-9 showed strong acid production against *P. aeruginosa* and *S. enterica*, the drop in pH was small against *C. koseri* and *K. pneumoniae*. The presence of *S. enterica* showed the highest medium acidification when exposed to the *W. paramesenteroides* strains, while other indicators caused the opposite (Figure 4). *P. aeruginosa*, however, gave a mixed picture. While isolate 22.4 7A-9 caused the highest acidification of all, co-culture with four other isolates resulted in mild acidification.

**Figure 5:**
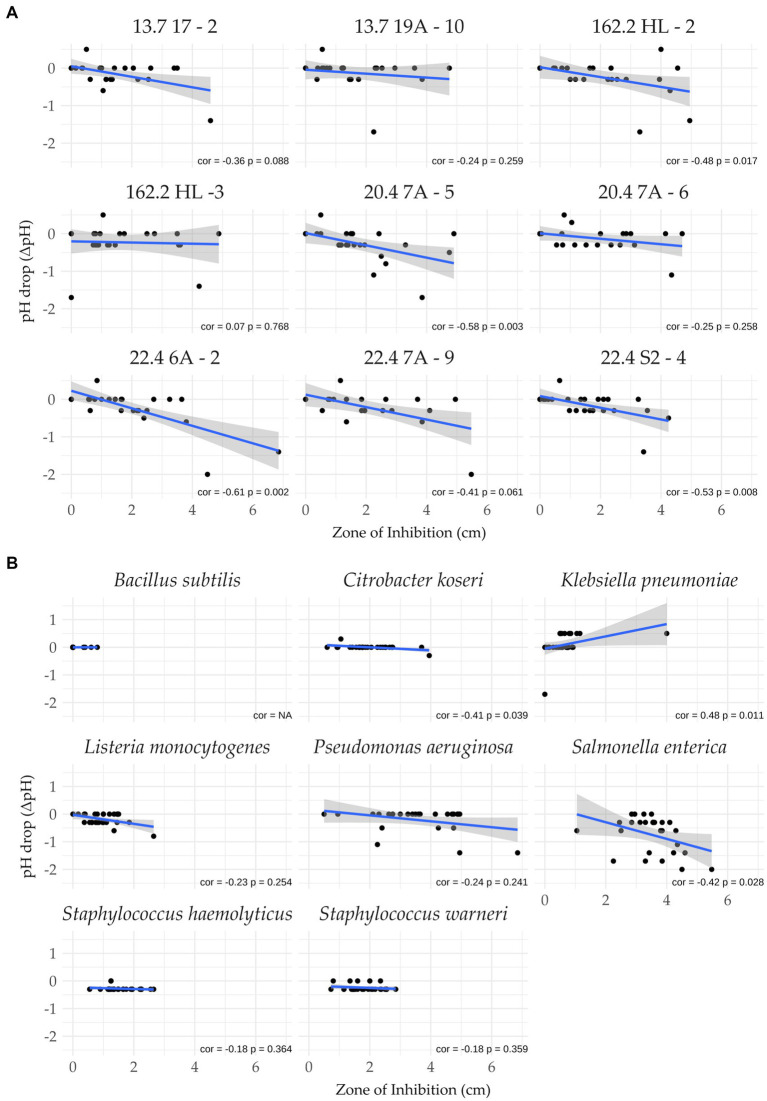
Pearson correlation analysis. Shown is the relation between pH drop and the sizes of zones of inhibition. **(A)** *W. paramesenteroides* isolates versus indicator strains and **(B)** indicator strains versus *W. paramesenteroides* isolates. cor, correlation coefficient; *p*-value (significance threshold <0.05).

To examine a relation between antimicrobial activity and acidification more rigorously, Pearson correlations were performed for each pair of strain and indicator. As shown in Figures 5A,B, significant negative correlations (cor − 0.48 to −0.61, *p* < 0.05) were observed for strains 05.3162.2 HL-2, 20–4 7A-5, 22.4 6A-2, 22.4 S2-4, indicating that acid production may cause an increase in the zone of inhibition (Figure 5A). For the remaining five isolates, it appeared that acid production played a minor role in causing antibacterial activity.

A second correlation analysis was conducted to calculate the behavior of the indicator strains regarding acid production (Figure 5B), and vice versa. It revealed that *S. enterica* (cor = −0.42, *p* = 0.028) was acid-driven, inhibited by the panel of isolates. That acidification did not significantly contribute to the antibacterial activity was found for *K. pneumoniae* (cor, 0.48; *p*, 0.011). The Pearson data on the remaining indicators showed no significant correlations.

**Figure 6:**
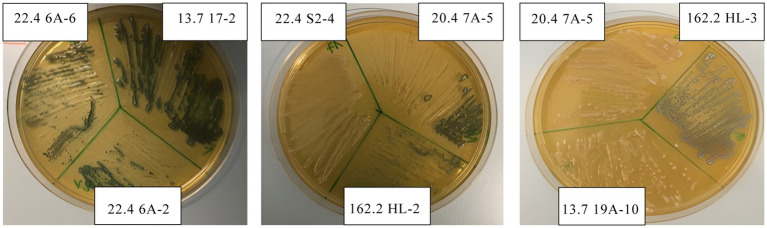
Production of hydrogen peroxide. Experiments were carried out in triplicate.

All 40 isolates were tested for their ability to produce H_2_O_2_, with 18 testing positive (data not shown). Within this group, six of the nine isolates selected for lawn-on-spot assays synthesized the antibacterial compound under the conditions used (Table 3 and Figure 6).

**Table 3:** Ranking of antibacterial efficacy.

Strain	Antibacterial efficacy ranking	Sum (cm)	Mean (cm)	Antibacterial efficacy correlation to acid production	Significant antibacterial correlation to acid production (*p < 0.05)	H_2_O_2_	Best performer against indicator
22.4 7A-9	1	18.75	2.68	−0.41	0.061	−	Bs, Ck, Pa, Se, Sw
162.2 HL-2	2	16.43	2.35	−0.48	0.017*	+	Ck, Kp, Sw
22.4 6A-2	3	15.88	2.28	−0.61	0.002*	+	Sh
20.4 7A-5	4	14.26	2.04	−0.58	0.003*	+	Lm
20.4 7A-6	5	14.10	2.02	−0.25	0.258	+	/
162.2 HL-3	6	13.03	1.86	−0.07	0.768	+	/
22.4 S2-4	7	12.44	1.78	−0.53	0.008*	−	/
13. 7 19A-10	8	12.39	1.77	−0.24	0.259	−	/
13.7 17–2	9	11.24	1.60	−0.36	0.088	+	/

Sum, added zones of inhibition; mean, average inhibition zone; Bs, *B. subtilis*; Ck, *C. koseri*; Kp, *K. pneumoniae*; Lm, *L. monocytogenes*; Pa, *P. aeruginosa*; Se, *S. enterica*; Sh, *S. haemolyticus*; Sw, *S. warneri*.

Table 3 shows the ranking of isolates according to their antibacterial efficacy. The 22.4 7A-9 strain performed best in five out of eight indicators. It appears that neither the production of organic acid nor H_2_O_2_ is a factor that contributes entirely to the antibacterial effect. In contrast, the second most effective isolate, 05.3162.2 HL-2, was found to produce both acid and H_2_O_2_. In summary, one-third of the isolates showed the formation of hydrogen peroxide in combination with significant acid production. This was not the case for the remaining six.

### 3.4 Genome analysis

**Figure 7:**
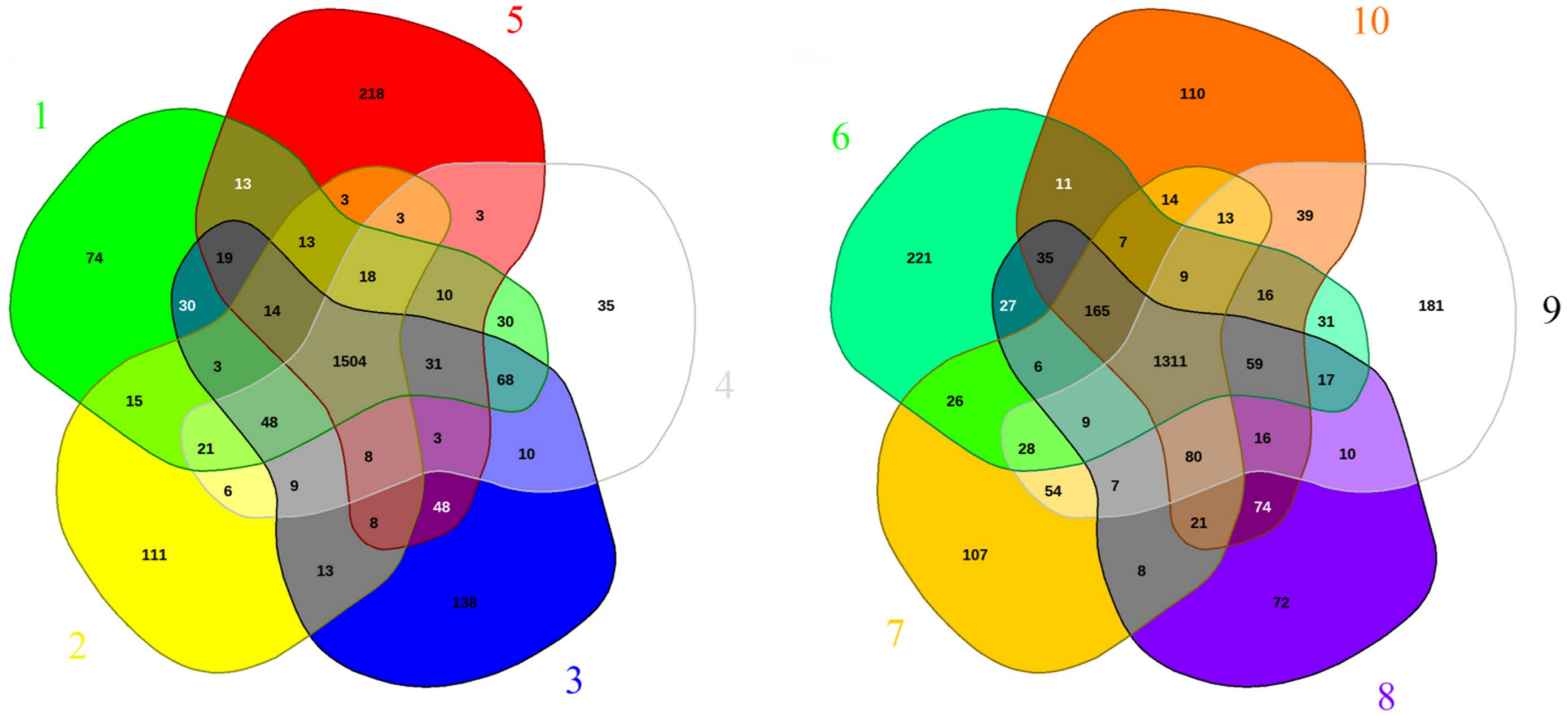
Pan-genomes of *W. paramesenteroides*. Ten completed genomes were divided into two sets for the shown genomic comparisons. The Venn diagrams illustrate the number of genes of the core genomes in the center of each group, surrounded by accessory genes between two to four genomes, and the number of singletons in the periphery. Strain numbering is as follows: 1, A47_1; 2, DSM 20288; 3, LCW-28; 4, STCH_BD1; 5, WP12; 6, MbWp_142; 7, W31; 8, FDAARGOS_414; 9, FL3; 10, JL_5.

The above-described experimental data show that *W. paramesenteroides* strains exhibited pronounced genetic variations according to RAPD analysis. These go hand in hand with differences in antibacterial efficacy, acidification, and H_2_O_2_ production. To gain more information on the genomic plasticity, we analyzed the available genomes of *W. paramesenteroides.* Therefore, only completed genome assemblies from the NCBI genome database were included (Table 4). The key data show a variation in genome length from 1.947.910 to 2.149.463 base pairs and in annotated genes ranging from 1,936 to 2,184, indicating a decent genomic plasticity. The EDGAR web server platform was used for genomic comparisons. Since the server limits genome comparisons to five at a time due to complexity, we divided them into two groups (Figure 7). The Venn diagrams show that the two sets shared 1,311 and 1,504 core genes, respectively. Since the genomes have about 2,000 annotated genes, roughly 500 genes per genome can be considered as accessory. To find out the number of unique genes (singletons), each genome was compared to the remaining nine. As shown in Table 4, the number of singletons ranged from 14 to 132, with an average of 52 and a total of 524. This means an additional, unique metabolic capacity between 2% and 6%.

**Table 4:** Key data of *W. paramesenteroides* genomes.

Strain	Source	Genome (Mbp)	Plasmids	Genes	Singletons	Accession
A47_1	*Bombyx mori*	2.080.060	1	2114	46	GCF_050613345.1
DSM 20288	Dairy	2.149.463	3	2147	77	GCF_051903945.1
FDAARGOS_414	Environment	1.947.910	0	1936	61	GCF_002386265.1
FL3	Fermented pepper	2.024.293	0	2069	46	GCF_028994215.1
JL_5	Moromi (soy sauce)	1.963.850	0	1952	14	GCF_045005335.1
LCW-28	Moromi (soy sauce)	1.990.966	2	1991	52	GCF_045006675.1
MbWp_142	Mulberry wine	2.007.410	1	2031	132	GCF_036327715.1
STCH_BD1	Ensiled sorghum	2.052.536	1	2072	36	GCF_015689215.1
W31	Idli batter	2.033.487	1	2039	27	GCF_030168775.1
WP12	Budu (fish sauce)	2.075.982	2	2,184	33	GCA_052281005.1

A search for bacteriocin-encoding genes present in the 10 genomes was conducted with the BAGEL4 web server, which provides the most comprehensive database of bacteriocin-encoding genes. No bacteriocin genes were detected. In addition, the peptide sequence of the only so far reported bacteriocin Weissellin A from *W. paramesenteroides* DX (UniProtKB/Swiss-Prot: B3A0N4.1) was screened for homologous proteins using the NCBI BLASTP server. The 43 amino acids of Weissellin A showed 73% overall sequence identity to leucocin A/sakacin P family class II bacteriocins from *L. mesenteroides* and other LAB. No homologs were found within the 61 available genomes of *W. paramesenteroides* strains. This showed that known bacteriocins are not present in *W. paramesenteroides*.

## 4 Discussion

Strains of *W. paramesenteroides* are frequently recovered from carbohydrate-rich niches such as raw milk, cheeses, fermented vegetables and cereals, and silages, consistent with their heterofermentative ecology (Fusco et al., 2015). The good growth characteristics of *W. paramesenteroides* strains, and the ubiquitous occurrence favors the use as probiotics, starters in food fermentation, and as protectives. This study aimed to investigate the antibacterial potential of *W. paramesenteroides* strains isolated from foremilk and teat canal biofilms. The data display a marked genetic plasticity, broad but varying antibacterial efficacies, differences in medium acidification depending on the confronted indicator strain, and H_2_O_2_ production in some isolated strains. Analysis of available genomes indicates that intraspecific genomic fluidity may underlie the observed phenotypic manifestations.

The data of RAPD analyses showed that 38 out of 40 isolates had less than 95% gene fragment pattern consistency and represented subspecies (Nielsen et al., 2014). Two pairs of isolates were clonal. The pair 27.4 5–4 and 27.4 6-1was isolated from foremilk samples of the same farm from cows number five and six. A third isolate, 27.4 8–1, from cow eight of the same farm was quite diverse. The second pair of clonal isolates, 13.7 22A-1 and 13.7 22A-2, was from the same teat canal biofilm of cow 22. However, we also isolated three subspecies, namely 27.8 11A-2, 27.8 11A-5, and 27.8 11A-6, from one teat canal of a cow from another farm. It also accounted for the isolates 20.4 7A-5 and 20.4 7A-6. It demonstrated that several subspecies can be present in one udder. We have recently characterized a set of 32 *Pediococcus pentosaceus* strains isolated from the same environment, in which data demonstrated that all isolates similarly represented subspecies (Fischer et al., 2025). Such genetic variations have been reported for *Lactobacillus plantarum* and for a set of more than a 1,000 *lactobacilli* isolated from raw cheese (Johansson et al., 1995; Rossetti and Giraffa, 2005). Three *B. subtilis* strains isolated from the sesame-flavored liquor Daqu also showed distinctly different amplification products in RAPD analysis experiments, indicating that similar genetic plasticity may be widespread among bacterial species (Wu et al., 2021).

All strains of *W. paramesenteroides* were able to inhibit the applied indicator organisms with the exception of *B. subtilis*. Strains 22.4 7A-9 and 05.3162.2 HL-2 were the best performers against five and three of eight indicators, respectively. In comparison to our previous study on *P. pentosaceus*, the findings corroborate that *W. paramesenteroides* and *P. pentosaceus* are able to defeat a broad range of target species (Fischer et al., 2025). While both species showed similar efficacies against some indicators, that is, *P. aeruginosa* or *L. monocytogenes*, *W. paramesenteroides* strains were clearly more efficient against *C. koseri* and *S. warneri* (Fischer et al., 2025). *Pabari et al.* have studied four other indicators with similar results, demonstrating broad antibacterial efficacies (Pabari et al., 2020). Nevertheless, *W. paramesenteroides* strains do not distinguish as they also inhibit many beneficial bacteria (Papagianni and Papamichael, 2011). This was shown by applying culture supernatants containing the bacteriocin Weissellin A (Papagianni and Papamichael, 2011).

When each isolate was exposed to each indicator strain, it became obvious that the acidification of the medium depended on the presence of the respective indicator strain. The highest drop in pH was detected when *W. paramesenteroides* and *S. enterica* were in co-culture, while almost none or even an increase in pH was found when *K. pneumoniae* or *B. subtilis* were applied. Among the *W. paramesenteroides* isolates, strains 05.3162.2 HL-2 and 22.4 7A-9 were the strongest acid producers. The responses of the indicator species suggest that they have different answers to acid stress (Guan and Liu, 2020; Zhou and Fey, 2020). A study of the transcriptome of *B. subtilis* cells that were stressed with the weak acids acetate and sorbate revealed that the *ureABC* operon, encoding urease, was up-regulated (Ter Beek et al., 2015). The enzyme is a major factor in coping with acid stress. It converts urea into two ammonia molecules that absorb protons, yielding ammonium (Ter Beek et al., 2015; Liu et al., 2020; Lin et al., 2022; Subramaniyan et al., 2023). The result is the buffering of acid. Ureases are also operative in the here used indicators *K. pneumonia*, *P. aeruginosa*, and *S. warneri*. Especially, *K. pneumoniae* strains are strong producers of urease, which is in good agreement with our data. Only one isolate caused medium acidification in co-culture with *K. pneumoniae* (Lin et al., 2022). The urease-negative indicators *S. enterica*, *S. haemolyticus,* and *L. monocytogenes* were less efficient against acidification. However, it should be noted that acid tolerance responses are multifaceted. Many genes have been identified, such as the synthesis of ammonia by the arginine deiminase pathway, amino acid decarboxylation systems, proton export by F_1_F_0_ ATPases, quorum sensing disruption or the involvement of global regulators, such as the carbon catabolite protein CcpA (Titgemeyer and Hillen, 2002; Ter Beek et al., 2015; Guan and Liu, 2020; Zhou and Fey, 2020; Roux et al., 2022; Rotta et al., 2025).

The production of H_2_O_2_ and thus the ability to exert oxidative stress was detected in about half of all isolates. The best performing isolate 27.4 7A-9 was not a producer of H_2_O_2_, indicating that other antibacterial mechanisms might be superior (Table 3). This raises the question of how the indicator strains might mask the full effect of hydrogen peroxide. The Gram-negative indicators used in this study possess various defense mechanisms to cope with reactive oxygen species. These are beside hydrogen peroxide, superoxide anions and hydroxyl radicals, which are able to primarily oxidize all RNA species, DNA, proteins, and cell membranes (Huang et al., 2013; Seixas et al., 2022). In *K. pneumoniae*, the catalases KatE, KatG, and peroxidases AhpC and GST neutralize H_2_O_2_ (Huang et al., 2013). They are triggered by the global regulator OxyR, which also stimulates the protective formation of biofilms and extracellular polysaccharides (EPS) (Hennequin and Forestier, 2009). This was shown by phenotype analysis of an *oxyR* deletion mutant, which is sensitive to H_2_O_2_ and unable to form biofilms (Hennequin and Forestier, 2009). For *S. enterica*, it has been shown that the catalases KatE and KatG are crucial for biofilm tolerance to H_2_O_2_, as their activity was reduced in the corresponding catalase mutant strains (Hahn et al., 2021). These catalases are also regulated by OxyR, which is itself activated by H_2_O_2_ (Pardo-Esté et al., 2018). Regarding *C. koseri*, defense mechanisms against oxidative stress have not yet been reported. We performed protein alignments with OxyR and KatG from *K. pneumoniae* and *S. enterica*, which show protein identities ranging from 83 to 97%, suggesting that *C. koseri* possesses very similar defense mechanisms. The OxyR regulon is also operative against oxygen stress in *P. aeruginosa*. Transcriptional analysis of the *katA* promoter and transcriptomics revealed that the expression of the *katA* gene, which encodes the major catalase, is mediated by OxyR in the presence of H_2_O_2_ (Palma et al., 2004; Heo et al., 2010). In *B. subtilis*, PerR is the main transcription factor involved in the expression of the catalase *katA* gene, the peroxidase-encoding ahpCF operon, and *mgrA* encoding a DNA-protecting protein (Bsat et al., 1996; Seixas et al., 2022). PerR binds as a metalloregulatory protein to ferrous iron (Fe^2+^) at a regulatory site. Increasing amounts of H_2_O_2_ oxidize the regulatory iron atom (Lee and Helmann, 2006). PerR-Fe^3+^ is released from its binding site, leading to derepression of *katA* and *ahpCF* (Seixas et al., 2022). In *L. monocytogenes* and in *staphylococci*, PerR regulates *katA*, *ahpCF, and mgrA*, as well (Horsburgh et al., 2001; Chen et al., 2006; Ruhland and Reniere, 2019; Cesinger et al., 2021).

The gene pool of *W. paramesenteroides* was described through genomic comparisons, revealing several hundred accessory genes and up to 6% singletons. The genome fluidity of *W. paramesenteroides* strains is comparable to that reported in our *P. pentosaceus* study (Fischer et al., 2025). A study of four genomes of *W. confusa* and another one, in which three complete and 39 draft genomes of *W. paramesenteroides* were analyzed, came to corroborating results (Yuan et al., 2021; Wan et al., 2023). Wan et al. (2023) reported the number of 1990 cloud genes that occurred in less than 15% of the *W. paramesenteroides* genomes.

The presence of bacteriocins from the genus *Weissella* remains rare (Leong et al., 2013; Fusco et al., 2015). Only one, Weissellin A, from *W. paramesenteroides* has been reported (Papagianni and Papamichael, 2011). It was able to inhibit many Gram-positive microorganisms but not *S. enterica*. Since the here examined strains were able to combat *S. enterica*, the mechanism must be independent of Weissellin A. Weissellin A showed high similarity to leucocin A/sakacin P family class II bacteriocins from *L. mesenteroides* and other LAB. It was, however, not detected in 61 *W. paramesenteroides* genomes according to BLASTP protein searches. Wan et al. reported that a pediocin-like bacteriocin is present in many *W. paramesenteroides* genomes (Wan et al., 2023). They concluded that the gene product may not have an antibacterial function, as strains encoding the pediocin-like gene did not inhibit *Micrococcus luteus*, which, however, is sensitive to Weissellin A (Wan et al., 2023). Thus, the currently available data suggest that *W. paramesenteroides* strains do not possess any known bacteriocins.

*W. paramesenteroides* strains have a repertoire of diverse mechanisms to combat competing bacteria. Our data suggest that the lowering of pH by organic acids is sufficient as long as the target bacterium is susceptible to acid stress. If not, other mechanisms become operative (Pabari et al., 2020; Singh et al., 2024; Thuy et al., 2024; Chaichana et al., 2025; Cui et al., 2025; Rotta et al., 2025). Although we did not sequence our isolates, the observed genome fluidity derived from RAPDs and public genomes of *W. paramesenteroides* strains points to the presence of strain-specific genes encoding antibacterial activity factors. To uncover these respective genes present in the pan-genome, further experiments will be necessary.

In conclusion, we support the suggestion of Ndagano et al. (2011) that the antibacterial activity is caused by different, synergistic acting mechanisms. It can be considered a multi-barrier system to meet diverse environmental conditions and exposure to diverse microorganisms.

## Data Availability

The raw data supporting the conclusions of this article will be made available by the authors, without undue reservation.
